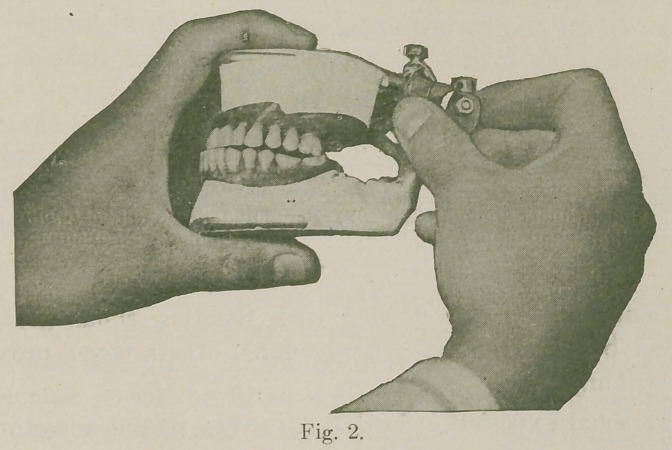# Antagonizing of Complete Artificial Dentures

**Published:** 1910-03-15

**Authors:** George H. Wilson

**Affiliations:** Cleveland, Ohio


### ANTAGONIZING OF COMPLETE ARTIFICIAL DENTURES.

BY GEORGE H. WILSON, D.D.S., CLEVELAND, OHIO.

The antagonizing of artificial denture is purely mechanical, therefore a laboratory operation. The expression, or esthetics, is developed with the patient in the chair.

There are certain lines in the ideal or normal arrangement of the teeth that must be observed in their temperamental modifications. These ideal lines are the arc of a circle described by the six anterior teeth, the straight, diverging line of the buccal surface of the bicuspids and molars, the compensating curve of the occlusal surface of the bicuspids and molars, and the lateral inclination of the teeth. The twelve anterior teeth are arranged in a straight occlusal line and the compensating curve is formed by the elevation of the teeth distal to the first molar.

To begin setting the teeth a portion of wax representing the upper teeth upon either side of the mouth should be removed bodily—that is, the segment of wax extending from the median and to the high lip line and either the left or the right side of the upper wax model. This leaves the wax contour of the gum portion intact. The six teeth, having been properly ground, are set in place to restore the contour of the removed wax. The incisors and cuspid should be given such a variation from the normal outline as the judgment of the dentist dictates; this arrangement, however, will be subject to change when the dentures are

Fig. 1.

tried in the mouth. The bicuspids and first molar are given such lateral inclination as is indicated by the pitch of the condyle path; but the morsal surfaces are arranged on a straight occlusal plane. These six teeth are secured with hard wax and the same procedure is applied to the opposite side. The twelve upper teeth are now mounted to a practically straight occlusal plane.

The lower wax rim, one side at a time, is cut away and the teeth are arranged in their relation to the upper ones. The bicuspids and molar must perfectly interdigitate, while the incisors and cuspid should be just free and but slightly underlap the upper ones. The teeth should now be placed in a lateral occlusion, and such corrections should be made as may be necessary. The six teeth on the opposite side are treated in the same manner. Twenty-four teeth have now been arranged in sections of six, and all are mounted to a straight occlusal plane (Fig 1). The wax securing these teeth to the base plate should be thoroughly cooled. The lower second molar upon either side is to be placed next. The placing of this one tooth forms the compensating curve. This tooth is easily located by cutting away the hardened wax in the location of the second molar and by supplying well-softened wax. The tooth should be placed at an angle parallel with the condyle path and in a little more elevated position than will be required when adjusted. The condyle path upon the side of the articulator on which the molar is being adjusted is grasped with the thumb and forefinger and both arms of the articulator are firmly held in the palm of the other hand. By repeatedly placing the teeth in the lateral and incisal occlusal positions, the second molar will be properly located in the softened wax (Fig. 2). It may be necessary with the thumb and finger to aid the direction of the tooth. It is apparent that the wax supporting the twenty-four teeth must be cold and hard while adjusting the second molar in the soft wax. The second lower molar upon the opposite side is mounted in like manner.

As the upper second molar can occupy but one position, all that remains to be done is to cut away sufficient wax to permit the tooth to drop into occlusion, and to make it fast with melted wax.

Is it not evident that in constructing a full upper and lower set of teeth, the only triangle that is necessarily given practical consideration is the small triangle formed by the

Fig. 2.

upper first molar and the lower first and second molars? The key to the correct anatomical antagonizing of complete dentures is represented by these three molars. If this conclusion be correct, then it follows that when the teeth are placed in incisal occlusion there can be no cusp contact except between the anterior teeth and the upper first molar and the lower second molar; because, if the twelve teeth in each dental curve or arch have a straight occlusal plane, and if one end be raised, there can be no contact except at the other end. If there is, however, much length to the cusps of the bicuspids, the upper cuspid may occlude with the lower first bicuspid when placed in incisal occlusion. This certainly is no disadvantage, for as long as there are three points, properly placed and in contact, there can be no tilting. As the teeth are coming in crushing contact progressively, far less power will be required than if all the teeth occlude at the same time. As only about one-fourth of the crushing force can be used with artificial dentures that may be used with the natural organs, any arrangement that economizes force is a blessing.—*The Summary*, March, 1909.

## Figures and Tables

**Fig. 1. f1:**
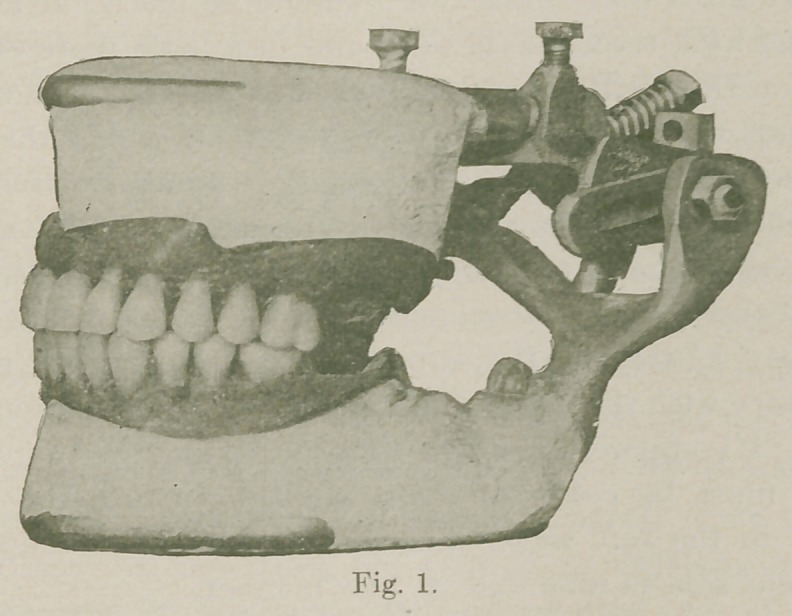


**Fig. 2. f2:**